# Domain-specific cognitive function in euthymic bipolar disorder: a systematic review and meta-analysis

**DOI:** 10.1017/S0033291725101827

**Published:** 2025-11-05

**Authors:** Samuel Swidzinski, Dimosthenis Tsapekos, Pricilla Swidzinska, Wenjia Zhang, Moxun Zheng, Edward Millgate, Rebecca Strawbridge, Roxanna Short, Ben Carter, Peter Gallagher, Jolanta Zanelli, Eugenia Kravariti, Abraham Reichenberg, Allan H. Young, Robin M. Murray, Sameer Jauhar

**Affiliations:** 1 Institute of Psychiatry, Psychology and Neuroscience, King’s College, London, UK; 2 University College London, London, UK; 3 Faculty of Education, University of Cambridge, Cambridge, UK; 4 Translational & Clinical Research Institute, Newcastle University, Newcastle, UK; 5 Department of Psychiatry, Icahn School of Medicine at Mount Sinai, New York, NY, USA; 6 Divsion of Psychiatry, Imperial College, London, UK

**Keywords:** bipolar disorder, cognitive functioning, meta-analysis, systematic review, euthymia

## Abstract

**Background:**

Euthymic bipolar disorder (BD) is associated with general and domain-specific cognitive impairment, which predicts poor occupational and social functioning.

**Methods:**

We searched Embase, Medline, and PsycInfo for articles published between database inception and June 2024, examining cognitive domains in euthymic BD. We conducted meta-analysis, meta-regressions, including premorbid IQ, demographic, and clinical variables. Newcastle Ottawa Scale, *I* ^2^ statistic, and funnel plots/Egger’s and Begg’s Test were used to assess quality, heterogeneity, and publication bias, respectively. The Benjamini-Hochberg (BH) procedure was utilised for multiple comparisons.

**Results:**

We identified 95 groups from 75 studies (*N* = 4,404 BD & 4,037 HC). BD showed significant impairment in general cognitive functioning (Hedge’s *g* = −0.58, 95%CI: −0.79, −0.37, *p* <.01), verbal memory (Hedge’s *g* = −0.70, 95%CI: −0.79, −0.60, *p* <.01), executive function (Hedge’s *g* = −0.69, 95%CI: −0.78, −0.60, *p* <.01), visuo-spatial memory (Hedge’s *g* = −0.68, 95%CI: −0.83, −0.53, *p* <.01), attention/processing speed (Hedge’s *g* = −0.64, 95%CI: −0.75, −0.54, *p* <.01), working memory (Hedge’s *g* = −0.61, 95%CI: −0.74, −0.49, *p* <.01), and premorbid IQ (Hedge’s *g* = −0.24, 95%CI: −0.36, −0.12, *p* <.01). Demographic and clinical factors were not associated with cognitive performance, except for a statistically significant, but small positive correlation between years of education and lower impairment in verbal memory, *β* = .066, adjusted *p* <.05.

**Conclusions:**

Our findings highlight cognitive domains impaired in euthymic BD, indicating targets for interventions. Substantial variance is unexplained, warranting focus on larger samples of individual-level data.

## Background

Bipolar disorder (BD) is associated with general and domain-specific cognitive impairment that extends to periods of euthymia (Cullen et al., 2016; Martínez-Arán et al., 2004). The prevalence of severe impairment in at least one cognitive domain is approximately 40% (Martino et al., 2008), though overall, there is a substantial discrepancy in the definitions and prevalence of cognitive impairment throughout the literature (Cullen et al., 2016). Domains such as verbal memory and attention appear particularly impaired (Bourne et al., 2013; Zanelli, 2012) and predict poor functional outcomes (Burdick et al., 2014; Hermens, Naismith, Redoblado Hodge, Scott, & Hickie, 2010; Jordan et al., 2018), including occupational and social functioning (Brissos, Dias, & Kapczinski, 2008; Thompson et al., 2005).

Delineating factors associated with cognitive impairment is relevant for identifying modifiable risk factors, which may represent treatment targets for interventions such as cognitive remediation (CR) (Strawbridge et al., 2021). Factors associated with cognitive impairment include duration of illness (Zanelli, 2012), number of episodes (López-Jaramillo et al., 2010), lithium use (Wingo, Wingo, Harvey, & Baldessarini, 2009), history of psychosis (Lahera et al., 2008), BD type (Dittmann et al., 2008), substance misuse (van Gorp, Altshuler, Theberge, Wilkins, & Dixon, 1998), and demographic factors (e.g., age, gender, educational level, premorbid IQ) (Carrus et al., 2010; Lewandowski, Sperry, Malloy, & Forester, 2014; Martino, Valerio, Szmulewicz, & Strejilevich, 2017).

Given the likely confounding effects of current mood episodes (King, Stone, Cleare, & Young, 2019), it is recommended to examine cognitive impairment in euthymic BD (Miskowiak et al., 2017; Thompson et al., 2005). An initial systematic review and meta-analysis that examined domain-specific cognitive performance in euthymic BD pooled data from 26 studies (689 BD and 721 healthy controls (HC)) and found executive function and verbal memory to be the most impaired domains (*d* ≥ 0.8) (Robinson et al., 2006). A subsequent individual patient data meta-analysis pooling data from 31 studies (1267 BD and 1609 HC) found the greatest impairment in Trail Making Test B (TMT-B, executive functioning; Reitan & Wolfson, 1992), followed by digit span backwards (working memory; Griffin & Heffernan, 1983), Verbal Learning Test (VLT, verbal memory; Elwood (1995); de Sousa Magalhães et al., 2012), Trail Making Test A (TMT-A, attention/processing speed; Bowie & Harvey, 2006), and Wisconsin Sorting Test (WCST, executive functioning; Jones, 2021) (Bourne et al., 2013). These impairments remain significant after controlling for age, gender, and premorbid IQ.

Bourne et al. (2013) tested the effect of six clinical predictors (number of depressive episodes, number of manic episodes, number of total episodes, number of depressive hospitalisations, number of manic hospitalisations and number of total hospitalisations) on each test. TMT-A was associated with the number of depressive hospitalisations and total episodes. The number of manic episodes was associated with VLT scores; the total number of hospitalisations was associated with TMT-B and WCST Categories. Psychotropic medication was not associated with cognitive impairment. The authors concluded that further longitudinal studies were required. Demographic factors (e.g., age and gender) have also been related to cognitive impairment in BD (Navarra-Ventura et al., 2021).

Similar to schizophrenia (Jonas et al., 2022; Murray, Bora, Modinos, & Vernon, 2022), controversy exists in BD as to whether cognitive impairment is explained by neurodevelopmental deficits, progressive decline following illness onset, or both (Burdick, 2022; Goodwin, Martinez-Aran, Glahn, & Vieta, 2008); this has relied on studies of cognitive functioning and neuroimaging in BD. The neurodevelopmental theory is supported by studies of undiagnosed family members (Sanches, Keshavan, Brambilla, & Soares, 2008) and cognitive deficits identified in the first episode (Bora & Pantelis, 2015; MacCabe et al., 2010; MacCabe et al., 2013). The neuroprogressive theory is predominantly supported by longitudinal analyses of cognitive functioning (Bora & Özerdem, 2017), and cross-sectional correlation with illness duration and number of episodes (López-Jaramillo et al., 2010; Zanelli, 2012). These inconsistent findings may be explained by the well-established cognitive heterogeneity observed at the population level (Burdick et al., 2014), possibly reflecting distinct patient subgroups with differential illness trajectories (Millett & Burdick, 2021). Hence, it is likely that both neurodevelopmental abnormalities and neuro-progressive decline underlie and explain cognitive outcomes for different patients. An alternative method for testing neurodevelopmental or neurodegenerative theories is through comparing premorbid IQ against other domains. Premorbid IQ appears intact (Lewandowski, Cohen, & Öngur, 2011) or less impaired than other domains (Valerio, Lomastro, & Martino, 2020) in BD at the population level, supporting a combination of neuroprogressive and neurodevelopmental explanations for cognitive deficits observed in BD.

### Aims and hypotheses

Our primary aim was to compare performance on general cognitive functioning, premorbid IQ, and domain-specific cognitive functioning (executive functions, verbal memory, working memory, visuo-spatial memory, attention/processing speed) between euthymic BD and HC. We hypothesised that comparative impairment in BD would be highest on average in attention and verbal memory and lowest in premorbid IQ, in accordance with prior literature (Bourne et al., 2013; López-Jaramillo et al., 2010).

Our secondary aim was to examine if premorbid IQ, demographic, and clinical factors explained performance differences in cognitive functioning between BD and HC. We hypothesised that lower premorbid IQ (Bourne et al., 2013), male gender (Navarra-Ventura et al., 2021), BD type 1 (BD1) (Dittmann et al., 2008), high hospitalisation rate (Levy, Medina, Manove, & Weiss, 2011), history of psychosis (Lahera et al., 2008), use of antipsychotics, benzodiazepines and anticonvulsants (Cañada et al., 2021; Torrent et al., 2011), no use of lithium (Sabater et al., 2016), number of episodes (Robinson et al., 2006), and illness duration (Frey et al., 2008; Martino, Samamé, Ibañez, & Strejilevich, 2015) would be associated with greater differences in cognitive test performance between BD and HC.

## Methods

### Protocol development and registration

This systematic review and meta-analysis were registered in the International Prospective Register of Systematic Reviews (PROSPERO; ID: CRD42021284784). The Preferred Reporting Items for Systematic Review and Meta-Analysis (PRISMA) were followed throughout the review (Supplementary Material A).

### Alterations to Prospero registration

Following registration, reviewers decided to focus specifically on cognitive functioning in BD, avoiding additions of schizoaffective disorder due to increasing volumes of data in both diagnoses, allowing for a separate focus in different reviews. Additionally, premorbid IQ was added as a focus, following clinical input on the relevance of this as a predictor of cognitive impairment.

### Search methods and strategy

Articles were systematically searched across Embase (1947–2024), Medline (1946–2024), and PsychInfo (1806–2024) in June 2024. No age or date restrictions were implemented. The Population Exposure Control Outcome and Study Design (PECO-S) model was used as a guide in the formation of the search strategy: (bipolar disorder OR manic depress*) AND (cognitive function* IQ OR cognitive performance OR cognitive decline). Searches were performed via Ovid. Backwards and forwards citation searches were conducted, and authors of known cohorts examining cognition in BD were contacted.

### Data collection

**Figure 1.**
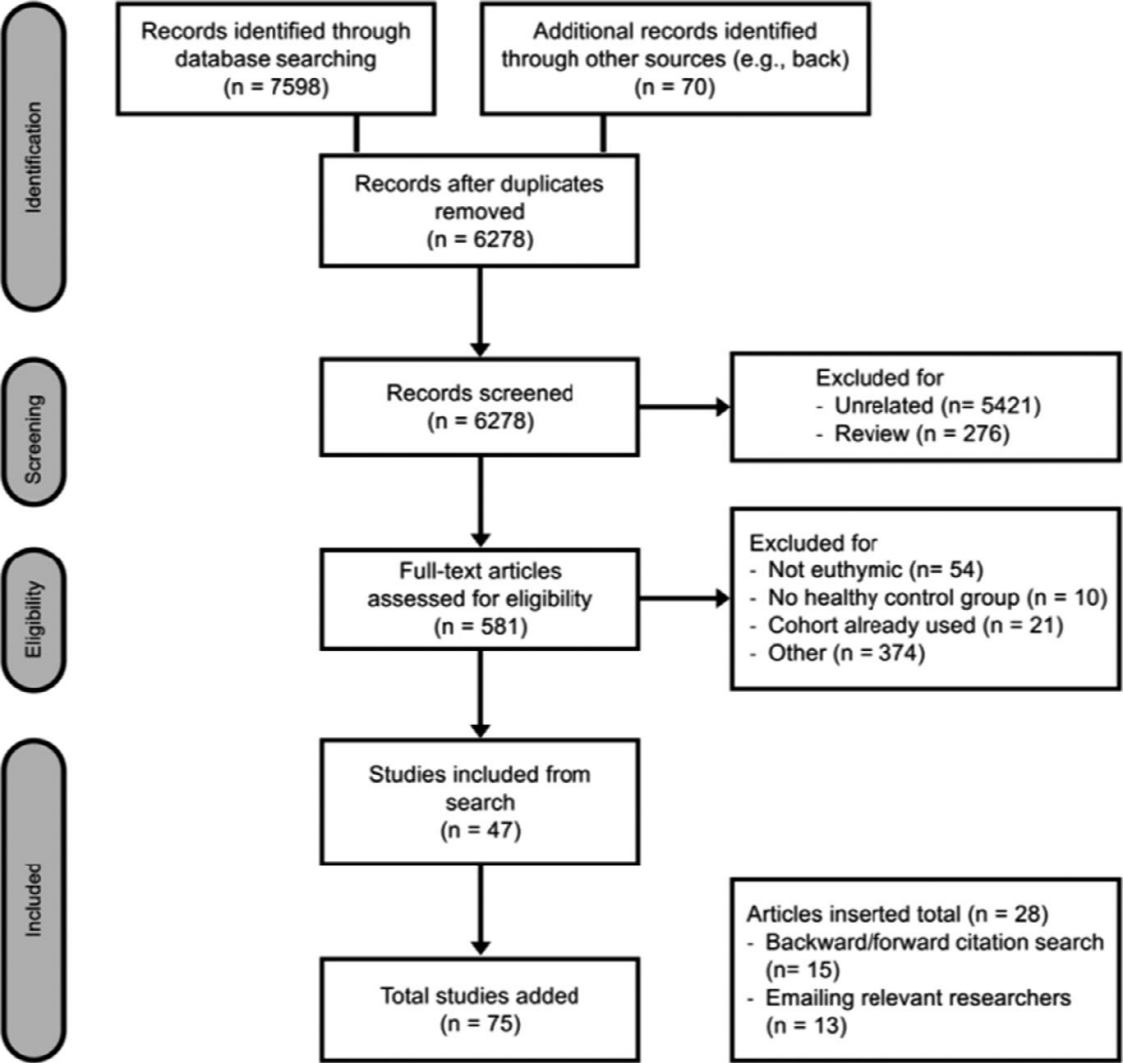
PRISMA flowchart.

The selection process occurred in two stages: (1) titles and abstracts were screened, and irrelevant articles were removed, and (2) full-text articles were then screened and selected for inclusion based on eligibility criteria (see Figure 1). Two reviewers conducted searches blind to the others’ decisions. The primary author (SS) searched for all studies, with co-authors screening studies before May 2021 (PS) and between May 2021 and June 2024 (WZ).

### Eligibility criteria

Inclusion criteria involved reporting of (1) euthymic BD and HC samples; (2) general, premorbid IQ (e.g., NART) or domain-specific cognition (e.g., verbal memory) data; (3) diagnosis using a structured interview or psychiatric assessment, either the Diagnostic and Statistical Manual for Mental Disorders (DSM-III, DSM-IIIR, DSM-IV, DSM-IVTR, DSM-5) or the International Classification of Diseases (ICD-9 or ICD-10), and (4) observational studies. Qualitative cognitive assessments, such as the executive interview (EXIT; Altshuler et al., 2007), were excluded from the review. Studies of any language were included and translated into English by native translators. Only baseline data from longitudinal studies were analysed.

### Quality assessment

Included studies were assessed for methodological quality using The Newcastle-Ottawa Scale (NOS) (Donnelly, Bracchi, Hewitt, Routledge, & Carter, 2017), which was completed independently by two reviewers (SS; MZ). Where there was no consensus, a third reviewer made the final decision (SJ). The NOS scale was completed as reported by previous researchers (Nayebirad et al., 2023). Cross-sectional and cohort studies were assessed and reported separately. A study was defined as ‘*very good*’ quality where scores ≥90%, ‘*good*’ quality where scores ≥70%, ‘*fair*’ quality where scores ≥50% and ‘*poor*’ quality where <50% (Nayebirad et al., 2023). The criteria for NOS in our study are shown in Supplementary Material B, with results in Supplementary Materials C and D.

### Data extraction

The following data was extracted from each study: name of the author, year of publication, diagnostic classification, geographical location, cognitive domain, cognitive battery, age of onset, duration of remission, duration of illness, BD1%, number of episodes, number of depressive episodes, number of hypomanic episodes, number of hospitalisations, history of psychosis, YMRS, HAM-D, medication choices (mood stabilisers, lithium, anticonvulsants, antipsychotics, antidepressants, benzodiazepines, and no medication). Sample size, age, ethnicity, sex, cognitive test scores, functioning test scores, and employment rates were recorded for BD and HC separately. Studies were allocated to one of three authors (SS; PS; WZ) for extraction, which was then checked for consistency by the other 2.

### Coding of cognitive batteries

Cognitive tests were grouped into one of seven categories: general cognitive functioning, premorbid IQ, executive functioning, working memory, verbal memory, visuo-spatial memory, and attention/processing speed. Coding of cognitive tests was performed based on previous studies of domain categorisation (Millgate et al., 2022) and in collaboration with four experts in neuropsychological assessment (AR; EK; PG; DT). Supplementary Material E gives a breakdown of cognitive tests.

When a study reported results for a specific domain (e.g., executive functioning) but did not mention the test used for domain results, data from the test were included within the function mentioned and labelled as ‘undefined’.

### Data synthesis

Fifty-six cognitive tests were grouped into the seven cognitive domains. Effect sizes for each cognitive domain were calculated for the mean difference between BD and HC cognitive performance in each study. Following this, a pooled estimate for Hedge’s *g* effect sizes between BD and HC was calculated for each domain.

The *metaset* command in STATA v17.0 (Viinikainen et al., 2022) was used to generate Hedge’s *g* effect sizes using a random mixed effects model for differences in cognitive performance between BD and HC groups (Hess, Quinn, Akbarian, & Glatt, 2015). Heterogeneity between included studies was assessed using the *I* ^2^ statistic, with high heterogeneity defined as *I* ^2^ > 75% (Higgins & Thompson, 2002). Publication bias was assessed through funnel plots and Egger’s and Begg’s test statistics (Montejo et al., 2022c).

Meta-regressions were conducted for specific risk factors (age, sex, age of onset, duration of remission, duration of illness, number of episodes, number of manic episodes, number of depression episodes, % BD1, number of hospitalisations, history of psychosis (yes/no), mood stabilisers (yes/no), lithium (yes/no), anticonvulsants (yes/no), antipsychotics (yes/no), antidepressants (yes/no) and benzodiazepines(yes/no)) in each cognitive domain, using the *metareg* command in STATA (Fatouros-Bergman, Cervenka, Flyckt, Edman, & Farde, 2014).

The Benjamini-Hochberg (BH) procedure (Bogdan, Ghosh, & Tokdar, 2008) was used to control for multiple comparisons in both primary analyses and meta-regressions (Van Haren, 2024).

Following expert input (BS; EM), a sensitivity analysis was conducted, removing studies that reported domain-specific scores (e.g., executive functioning) whilst not mentioning the specific tests that contributed to that score (e.g., TMT-B).

## Results

### Search and selection process

The flowchart in Figure 1 outlines the study selection process. Seventy-five observational studies were included in the review and meta-analysis. Relevant studies were removed due to not being limited to euthymic cases (Hidese et al., 2023; Juselius, Kieseppä, Kaprio, Lönnqvist, & Tuulio-Henriksson, 2009; Zanelli, 2012) and one study, which measured cognitive functioning through a qualitative measure (i.e., EXIT interview; Altshuler et al., 2007).

Ten studies reported on multiple groups. Specifically, Navarra-Ventura et al. (2021) reported male and female groups separately; Soni, Singh, Shah, and Bagotia (2017)), Martino et al. (2014), and Czepielewski et al. (2015) reported low and high functioning separately; Lahera et al. (2008) and Bora et al. (2007) reported psychotic and non-psychotic; van Gorp et al. (1998) reported alcohol dependence and no-alcohol dependence; Dittmann et al. (2008) reported BD1 and BD2; Rosa et al. (2014) reported four levels of functioning, ranging from high functioning to being unable to maintain personal self-care; Hasse-Sousa et al. (2024) reported with and without suicide attempts; Jones et al. (2023) reported cognitively impaired and not; Yang et al. (2024) reported drug-naive and long-term medication. In total, 95 groups were included, with 4404 BD and 4037 HC included in the meta-analysis. As each group included data from multiple cognitive tests, a total of 349 effect sizes were calculated: 16 groups included data on general cognitive functioning, 32 pre-morbid IQ, 83 executive functioning, 52 working memory, 65 verbal memory, 19 visuo-spatial memory, and 82 attention/processing speed.

### Characteristics of included studies

Tables 1 and 2 present demographic and clinical information regarding each study, respectively.

**Table 1.** Demographic characteristics of studies

Study	Group	N (BD/HD)	Sample Type (Location)	Age Range (Mean BD/HD)	%Female (BD/HC)	Education Years (BD/HC)	Cognitive Domains Tested
Navarra-Ventura et al. (2021)	Male	30/20	Outpatient (Spain)	18–64 (46.9/45.6)	100/100	11.4/11.4	Premorbid
	Male	30/20	Outpatient (Spain)	18–64 (47.5/46.1)	0/0	11.7/11.7	
Valerio et al. (2020)			Outpatient (Argentina)	18–65 (43.9/68.4)	68.4/64	14.4/13.7	Premorbid, executive, verbal, attention
Masuda et al. (2020)		30/30	(Japan)	(50.8/52.2)	50/43/3		General, executive, working, attention
Boland et al. (2015)		24/24	(Austria)	18–65 (32.6/31)	62.5/58.3	14/14.5	General, executive, working, attention
Frajo-Apro (2020)		29/79	University Sample (Turkey)	18–65 (45.9/44.6)	50/58.8	13/14.5	Premorbid, executive, working, verbal
İlhan (2018)		33/35	University Sample (Austria)	18–65 (36.9/37.8)		11.8/12.9	Premorbid, executive, working, verbal attention
Martino, Marengo, Igoa, and Strejilevich (2018)		56/30	Outpatient (Argentina)	50+ (63.7/65.1)	68.2/80	12.3/12.0	Premorbid, executive, working, verbal attention
Arslan, Tiryaki, Sarioğlu, and Çankaya (2017)		36/38	Outpatient (Turkey)	18–60 (38/37.9)	61.1/52.6	10.9/10.3	Executive, verbal, attention
Soni et al. (2017)	Low Functioning	30/30	Outpatient (India)	18–55 (32.9/31.7)	37/40	8.6/8.9	Executive, working, verbal, attention
	High Functioning	31/30	Outpatient (India)	18–65 (34.4/31.7)	42/40	8.5/8	
Jensen, Knorr, Vinberg, Kessing, and Miskowiak (2016)		193/110	Clinics and online advertisement (Denmark)	18–65 (36/35)	62/57	15/16	Executive, working, verbal, attention
Fernandes et al. (2016)		23/27	Outpatient (Brazil)	18+ (36/35)	30.4/37	13/14	General
Yang et al. (2014)		164/164	Outpatient (China)	(30.9/30.9)	57.9/57.9	11.5/11.2	Executive, attention
Suwalska and Łojko (2014)	Female	35/35	Outpatient (Poland)	26–75 (53.9/55.6)	100/100	13.4/13.1	Executive, visuo-spatial, attention
	Male	24/24	Outpatient (Poland)	26–75 (50/48.5)	0/0		
Zhou et al. (2013)		47/47	(Hong Kong)	16–50 (28.7/28.7)		14.3/15.5	Executive, working
Baysal et al. (2013)		60/20	Outpatient (Turkey)				Executive, verbal, attention
Ibanez et al. (2012)		13/25	Outpatient (Argentina)	18–54 (40.1/35.1)	38.5/36	16.5/17.2	Executive, verbal, attention
Normala et al. (2010)		40/40	Outpatient (Malaysia)	18–60	52.5/75		Executive, working, attention
Aydemir and Kaya (2009)		38/19	University (Turkey)	18–65 (38.5/35.5)	42.1/26.3		General, verbal, attention
Wobrock et al. (2009)		18/23	University (Germany)	42.1/30.1	61.1/56.5		Executive, working, verbal, visuo-spatial, attention
Lahera et al. (2008)	Psychotic	42/48	Lithium outpatient clinic (Spain)	18–70 (45.8/46.6)	66.7/66.6		Executive, attention
	Not-psychotic	33/48	Lithium outpatient clinic (Spain)	18–70 (51.2/46.6)	66.6 /51.2		
Trivedi et al. (2007)		15/15	Outpatient (India)	18–45 (34.4/34.3)	18/20	11.5/108	Executive, attention
Ozdel, Karadag, Atesci, Oguzhanoglu, and Cabuk (2007)		27/22	Outpatient (India)	20–55 (31.8/34.1)	70.4/77.3	10/10.4	Executive, working, verbal, visuo-spatial, attention
Krabbendam et al. (2000)		21/22	University (Netherlands)	< 60 (47.7/41.1)	77.3/54.5	4/4	General, executive, verbal, attention
van Gorp et al. (1998)	Alcohol dependent	12/22	Outpatient (USA)	(52.9/51.7)		15.2/15.0	Premorbid, attention, verbal, visuo-spatial, attention
	Not Alcohol dependent	13/22	Outpatient (USA)	(51.8/51.7)		15.9/15.0	Premorbid, executive, verbal, visuo-spatial, attention
Zubieta, Huguelet, O’Neil, and Giordani (2001)		15/15	Outpatient (USA)	(39/39)	40/40	16/16	General, executive, verbal, visuo-spatial, attention
Dittmann et al. (2008)	BD1	65/62	Outpatient (Germany)	(39.2/47)	46.2/56.5	11.8/11.6	Premorbid, executive, verba, visuo-spatial, attention
	BD2	38/62	Outpatient (Germany)			11.8/11.6	
Martínez-Arán et al. (2004)		40/30	Hospital (Spain)	20–60 (38.5/38.9)			Premorbid, executive, working, attention
Brissos et al. (2008)		55/50	Hospital (Portugal)	17–63 (37.1/35.4)	60/68	11.2/12.4	Premorbid, executive, working, visuo-spatial, attention
Bora et al. (2007)	Psychotic	40/30	(Turkey)	18–55 (38.7/38.3)	50/53	11.2/12.5	Executive, verbal, attention
	Not psychotic	25/30	(Turkey)	18–55 (36.8/38.3)	40/53	12.5/12.5	
Cavanagh, Van Beck, Muir, and Blackwood (2002)		20/20	(Scotland)	18–70 (43.6/50.0)	50/50		Premorbid, executive, verbal, attention
Clark et al. (2002)		30/30	(England)	18–60 (35.9/37.6)	43.3/46.7	16.2/15.6	Premorbid, executive, verbal, visuo-spatial, attention
Dias et al. (2009)		65/50	Outpatient (Spain)	17–55 (37.8/33.6)	64.1/72	10.9/13	Premorbid, executive, visuo-spatial, attention
Torrent et al. (2011)	Quetiapine	12/35	(Spain)	18–65 (45.6/39.1)	75/62.9	14.1/12.9	Executive, working, verbal, attention
	Olanzapine	26/35	(Spain)	18–65 (41.2/39.1)	36.4/62.9	13.7/12.9	
	Risperidone	30/35	(Spain)	18–65 (38/39.1)	37.9/62.9	13.1/12.9	
	Unmedicated	16/35	(Spain)	18–65 (42.1/39.1)	43.8/62.9	12.9/12.9	
El-Badri, Ashton, Moore, Marsh, and Ferrier (2001)		29/26	(England)	18–40 (30.7/27.7)	19/12		Executive, working, attention
Cheung, Halari, Cheng, Leung, and Young (2013)		52/52		18–64 (38.6/37.8)	63.5/38.6	12/14.04	
Czepielewski et al. (2015)	High function	17/28	(Brazil)	18–65 (40.7/32.5)	23.5/50	9.8	Verbal
	Low functioning	14/26	(Brazil)	18–65 (52.1/49.7)	35.7/50	11.39	
Gildengers et al. (2007)		20/40	(USA)	60+ (73.6/69.9)		15.7/13.6	Executive, verbal visuo-spatial, attention
Martino et al. (2008)		20/20	(Argentina)	60+ (66.6/70.5)	75/66	11.1/11.7	Premorbid, executive, verbal, attention
Martino et al. (2014)	Cognitively impaired	30/40	(Argentina)	18–60 (41.9/40.3)	66.7/70	14.1/13.9	Premorbid, executive, working, verbal, attention
	Cognitively preserved	30/40	(Argentina)	18–60 (38/40.3)	63.6/70	15/13.9	
Rosa et al. (2014)	Functioning=prior to bipolar	16/43	(Brazil)	18+ (41.8/45.7)	75/55.8	9.9/9.6	Working, verbal, attention
	Residual symptoms	11/43	(Brazil)	18+ (46.1/45.7)	81.8 /55.8	9.5/10.6	
	Social and occupational dysfunction	13/43	(Brazil)	18+ (52.6/45.7)	69.2/55.8	7.3/10.6	
	In need of care	14/43	(Brazil)	18+ (52.1/45.7)	71.4/55.8	8.3/10.6	
Schouws, Zoeteman, Comijs, Stek, and Beekman (2007)		15/15	(Netherlands)	60+ (73.1/72.5)	53.3/53.3	5.5/5.5	Premorbid, executive, working, attention
Schouws et al. (2009)	Early Onset	59/78	(Netherlands)	60+ (68.4/71.9)	53/72	5.2/5.3	Premorbid, executive, working, verbal, attention
	Late Onset	60/78	(Netherlands)	60+ (72.3/71.9)	52/72	4.9/5.3	Premorbid, executive, working, verbal, attention
Zaki, El-Batrawy, El-Missiry, Ibrahim, and Abdel-Aziz (2014)		30/30	(Egypt)	18–50 (25.4/25.8)		14.7/13.9	General, executive verbal, attention
Delaloye et al. (2011)	Baseline	15/15	(Switzerland)	50+ (67.9/68.3)		12.7/13.5	Working, attention
Mora, Portella, Forcada, Vieta, and Mur (2013)	Baseline	28/26	Lithium clinic (Spain)	18–65 (41.7/41.4)	50/46.2	10.1/12.2	Executive, working, verbal, visuo-spatial, attention
Santos et al. (2014)		62/40	Outpatients (Spain)	18–55 (44.4/44.8)		12.2/14.8	Executive, working, verbal, visuo-spatial, attention
Smith, Muir, and Blackwood (2006)	Baseline	21/33	Psychiatric clinic (Scotland)	(22.4/22.2)	66.8/57.6	16.9/17.3	Premorbid, executive, verbal, attention
Rossetti et al. (2022)		127/134	Outpatients (Italy)	>18 (44.5/41.5)	59.8/59.7	13.2/16.3	General, executive, working, verbal, attention
Joachimiak, Jaracz, and Jaracz (2022)		33/32	University (Poland)	18–65 (39.5/41.6)	67/47		
El Nagar et al. (2022)		20/20	Outpatients (Egypt)	18–60 (23.3/26.1)	40/40	12.2/12.8	Executive, attention
Gupta et al. (2022)		25/20	Outpatients (India)	18–45 (29.9/30.9)	24/25		Executive, working, verbal, visuo-spatial, attention
Chen et al. (2023)		34/35	Outpatients (China)	31.88/30.6	52.9/48.6	21.2/12.6	Executive, working, attention
Hasse-Sousa et al. (2023)		172/167	Outpatients (Brazil)	18–75 (48/44.7)	70.3/68.1	10.4/13.3	Verbal
Sonkurt, Altınöz, Danışman Sonkurt, and Köşger (2022)		19/21	Outpatients (Turkey)	(42/38.4)	36.8 23.8	14.1/12.5	Executive, working, visuo-spatial, attention
Martins et al. (2023)		31/91	Outpatients (Brazil)	18–70 (45.1/46.2)	64.5/78.7	9.8/15.3	General, verbal
Chang et al. (2022b)		60/66	Outpatients (Taiwan)	20–70 (39.9/35.5)	60/59.1		Executive
Montejo et al. (2022)		138/73	Outpatients (Spain)	>50 (59.5/61.7)	53.6/63	13.3/14.3	Premorbid, executive, working, verbal, visuo-spatial, attention
Yamaguchi et al. (2022)		5379	Outpatients (Japan)	(42.5/40.4)	35.9/21.5	15.3/16.6	Executive, attention
Chang et al. (2022a)		29/34	Outpatients (Taiwan)	18–70 (35.9/34.2)	61.3/61.3	14.9/16.2	Executive
Reininghaus et al. (2022)		56/53	Outpatients (Austria)	(39.8/37)	48.2/69.8		Premorbid, executive, verbal, attention
Karademir, Beyazyüz, Beyazyüz, Yılmaz, and Albayrak (2024)		41/40	Outpatients (Turkey)	18–50 (36.07/35.43)	0/0		Working
Chang et al. (2024)		44/40	Outpatients (Taiwan)	20–70 (38.8/33.5)	63.6/55	14.3/16.5	Executive, attention
Hasse-Sousa et al. (2024)	With suicide attempts	49/205	Outpatients (Brazil)	>18 (50.8/36.9)	77.6/78.5	10.9/15.1	Executive, working, verbal, attention
	Without suicide attempts	52/205	Outpatients (Brazil)	>18 (46.6/35.9)	61.5/78.5	10.9/15.1	
Selahaddin et al. (2024)		64/64	Outpatients (Turkey)	(33.2/31.4)	54.7/43.8		Executive, attention
Ciftci, Farhad, Metin, and Tarhan (2024)		169/45	Outpatients (Turkey)	18–70 (44.5/40.9)	46.2/35.6		Executive, working, verbal, visuo-spatial, attention
Lloyds, Chidambaram, Karthik, and Swathik (2024)		30/30	Outpatients (India)	18–45 (32.6/32.9)	42.9/33.3		Executive, working, verbal, visuo-spatial, attention
Leser et al. (2023)		86/93	Outpatients (Austria)	18–76 (45.2/37.8)	45.3/68.8		Premorbid, executive, verbal, attention
Quinlivan et al. (2023)		26/24	Outpatients (Germany)	18–65 (44.8/39)	50/67	16.0/15.4	General, executive, working, verbal, attention
Jones et al. (2023)	Young	70/153	Outpatients (Canada)	18–49 (27.4/28.1)	61.4/50.9	14.8/15.6	Executive, working, verbal, attention
	Old	48/44	Outpatients (Canada)	50–86 (64.4/66)	62.5/54.5	15.1/14.8	
Javadi, Shafikhani, and Yazdi (2023)		60/60	Outpatients (Iran)	18–60 (38.7/36.2)	46.7/50		Executive, working, verbal, attention
Løchen et al. (2023)		54/148	Hospital and outpatient (Norway)	>18 (33.8/39.9)	64.8/44.5	14.5/14.9	General
Fortea et al. (2023)	Cognitively impaired	83/50	Mental health services (Denmark)	18–65 (36/31.5)	64/63	13.8/16.1	IQ, Premorbid
	Cognitively normal	61/50	Mental health services (Denmark)	18–65 (30.5/31.5)	74/63	15.0/16.1	
Kjærstad, Søhol, Vinberg, Kessing, and Miskowiak (2023)	Baseline	266/190	Outpatients (Denmark)	15–70 (31.2/31.4)	65/62	15.0/16.1	Premorbid, executive, verbal, attention
Yang et al. (2024)	Drug-naive	57/50	Outpatients (China)	15–50 (22.7/24.2)	100/100	14.1/16.1	General, visuo-spatial, attention
	Long-term medication	64/50	Outpatients (China)	15–50 (24.1/24.2)	100/100	14/16.1	
Knorr et al. (2024)	Baseline	85/44	(Denmark)	18–60	48.2/43.3		General, executive, verbal, attention

**Table 2.** Illness type, severity, and functioning of participants in each study group

Study	Sample	%BD1	DOI (months)	Age of onset	No. of episodes (dep/manic)	Hospitalisations	% History of Psychosis	FAST (BD/HC)	GAF (BD/HC)	% Unemployed (BD/HC)
Navarra-Ventura et al. (2021)	Male		235.2	28.3	4.2/2.5	1.7	56.7			
	Female				5.7/1					
Valerio et al. (2020)		38.2	117	29.2	3.9/2.8					
Masuda et al. (2020)		76.7	236.4	31.1	8.9/unknown					
Boland et al. (2015)							44.4			
Frajo-Apor et al. (2020)		44.4	169.2							
İlhan, Demirel, and Şentürk-Cankorur (2018)		0					25.8	7/4.7		
Martino et al. (2018)		30	289.9						77.8/86.8	
Arslan et al. (2017)				25.3	2.8/2.8					
Soni et al. (2017)	Low Functioning		136.8	21.5	4.9/4.3	1.8	66.7		50.5/Unknown	
	High Functioning		135.6	23.1	2.2/3.5	1.3	61.3		83.1//unknown	
Jensen et al. (2016)		58	180		3/3					
Fernandes et al. (2016)		100								
Yang et al. (2014)		100								
Suwalska and Łojko (2014)	Male		256.8	28.4						
	Female		266.4	31.7						
Zhou et al. (2013)										
Baysal et al. (2013)			145.56	26.3						
Ibanez et al. (2012)		0								
Normala et al. (2010)		100	131.4	21						60/40
Aydemir and Kaya (2009)		86.8	154.8	26.6						
Wobrock et al. (2009)										
Lahera et al. (2008)	Psychotic		238.8	26.3	3.6/4.6	2.6				
	Not psychotic		254.4	30	5.3/4.5	2				
Ozdel et al. (2007)			114.6	22.6			40.7			
Trivedi et al. (2007)			44.4				40.7			
Krabbendam et al. (2000)			160.8	32.2	Unknown/3.9					
van Gorp et al. (1998)	Alcohol dependent		350	25.3	6.3/4.8					
	Not alcohol dependent		267.7	27.2	7.8/10.2					
Zubieta et al. (2001)		100	156	27	2.7/2.9	5.3	100		67.7/Unknown	
Dittmann et al. (2008)	BD1	100	177.8	24.3			72			
	BD2	0	233.5	28			26			
Martínez-Arán et al. (2004)			180	23.6		2.6			67.7/Unknown	
Brissos et al. (2008)		100	143.4	24.7	6.5/6.5	1.7	56.4			
Bora et al. (2007)	Psychotic	100	187.3	23.2	3/4.7	2.2	100			30/24
	Not psychotic	100	164.1	23.6	4.8/3.9	1.1	0			28/24
Cavanagh et al. (2002)			192	27.7	6/6	5.4				
Clark, Iversen, and Goodwin (2002)		100								
Dias et al. (2009)		100								
Torrent et al. (2011)	Quetiapine	100	190.8	27.5	6.2/5.7	2	33.3			72.4/unknown
	Olanzapine	66.7	188.4	25.7	8.2/12.1	3.1	71.4			36.4/unknown
	Risperidone	77.3	183.6	22.2	3.7/4.3	2.6	81.5			27.9/unknown
	Unmedicated	89.7								
El-Badri et al. (2001)		100	111.6	20.9						
Cheung et al. (2013)		100	159.6	24.6	5.1/5.2	4.2				42.3/0
Czepielewski et al. (2015)	High functioning		130.6							
	Low functioning		252							
Gildengers et al. (2007)		70								
Martino et al. (2008)		20	336	39.7		0.94			73.6/87.5	
Martino et al. (2014)	Cognitively impaired	56.7	142.1	29.5	3.46/3.39	0.64	50		74.2/90.4	
	Cognitively preserved	36.7	117.1	27.3	3.25/2.5	0.2	36.7		87/90.4	
Rosa et al. (2014)	Functioning=prior to bipolar	100	108			2		16.9/9.2		18.7/0
	Residual symptoms	100	132			1		25.7/9.2		27.3/0
	Social and occupational dysfunction	100	312			4.5		35.4/9.2		23/0
	In need of care	100	216			3		44.9/9.2		14.3/0
Schouws et al. (2007)		100	475.9	33.5	9/5	5.9	46.7			
Schouws et al. (2009)	Early Onset	64		27.6	5.5/7.4	4				
	Late Onset	77		53.8	3.2/3.4	2.6				
Zaki et al. (2014)		0	42.2							
Delaloye et al. (2011)	Baseline	50		34.1						
Mora et al. (2013)	Baseline	67.9	221.2	22.4	Unknown/2.5	3	78.6		72.3/92.4	
Santos et al. (2014)	Baseline		216	26.5	7.5/5.8	3.2				
Smith et al. (2006)				15	4.7/unknown					
Rossetti et al. (2022)		70.08	49.4	27.07		2.98			68.8/87.1	
Joachimiak et al. (2022)			74.4							45/0
El Nagar et al. (2022)		100	163.8							70/25
Gupta et al. (2022)			125.3	19.5						55/44
Chen et al. (2023)		71.3			3.5/1.7					
Hasse-Sousa et al. (2023)								26.1		13.7
Sonkurt et al. (2022)		100		24.7			47.37			
Martins et al. (2023)		187.2	29.3					26.1		
Chang, Chen, et al. (2022b) (None of the variables reported)										
Montejo, Jiménez, et al. (2022)		61.6	318.8	32.8	6.8/2.8	1.74	59.3	25.2		
Yamaguchi et al. (2022)		38	149.8							
Chang, Tseng, et al. (2022a) (None of the variables reported)										
Reininghaus et al. (2022)		67.3								
Karademir et al. (2024)		75.6		24						
Chang et al. (2024)		56.8								
Hasse-Sousa et al. (2024)	With suicide attempts		205	33.3				29.4		16.3/0.9
	Without suicide attempts		160.4	33.3				19.5		11.5/0.9
Selahaddin et al. (2024)		70.3								
Ciftci et al. (2024)		100								
Leser et al. (2023) (None of the variables reported)										
Lloyds et al. (2024)		46.6								
Quinlivan et al. (2023)		57.7	245.9	24.32	11.5/9.17	2.42				
Jones et al. (2023)	Young		97.1	19.29	6.2	1.3	70			
	Old		407	30.46	19.2	5.3	35.4			
Javadi et al. (2023)		100	159.1							
Løchen et al. (2023) (None of the variables reported										
Fortea et al. (2023)	Cognitively impaired	180.5								
	Cognitively normal	114.8								
Kjærstad et al. (2023)		96.6	22.6	11.45	0.87			17.9		
Yang et al. (2024)	Drug-naive		27.5	19.9						
	Long-term medication		64.4	18.7						
Knorr (2024) (None of the variables reported)	Baseline									

#### Demographic

53.47% of BD (SD = 17.63) and 52.92% (SD = 15.34) of HC were female. The mean age was 42.53 (SD = 11.03) and 41.04 (SD = 11.40) for BD and HC, respectively, while the mean number of education years was 13.00 (SD =5.42) for BD and 13.21 (SD = 2.75) for HC.

#### Illness severity

The mean and median duration of illness (in months) were 181.97 (SD = 83.44) and 173.10 (Q1 = 130.98, Q3 = 234.36, IQR = 103.38), respectively. The mean age of BD onset was 26.83 years (SD = 5.71). The mean number of episodes was 9.47, with 6.46 (SD = 4.38) depressive episodes and 4.66 (SD = 3.51) (hypo)manic episodes. The mean number of hospitalisations and percentage of participants with a history of psychosis were 2.49 (SD = 1.50) and 53.34% (SD = 24.77), respectively.

#### Medication

8.56% of BD participants were taking no psychotropic medication, 66.24% were on mood stabilisers, 50.69% on lithium, 47.49% on anticonvulsants, 50.25% on antipsychotic medication, 24.04% on benzodiazepines, and 30.27% on antidepressants.

#### Functional outcome

38.20% of BD were unemployed, compared to 22.01% of HC. Functioning Assessment Short Test (FAST) scores (*M* = 24.92, SD = 9.98) indicated a moderate level of functional impairment in BD.

### Quality assessment

Three of the 75 studies included were of very good quality, 46 of good quality, 23 of fair quality and three of poor quality, with low sample size, comparability between BD and HC in age and years of education, and absence of structured interviews being the primary reason for reduced quality. Supplementary Materials C and D present a detailed breakdown of the quality assessment of cross-sectional and longitudinal studies, respectively.

### Meta-analysis

**Figure 2.**
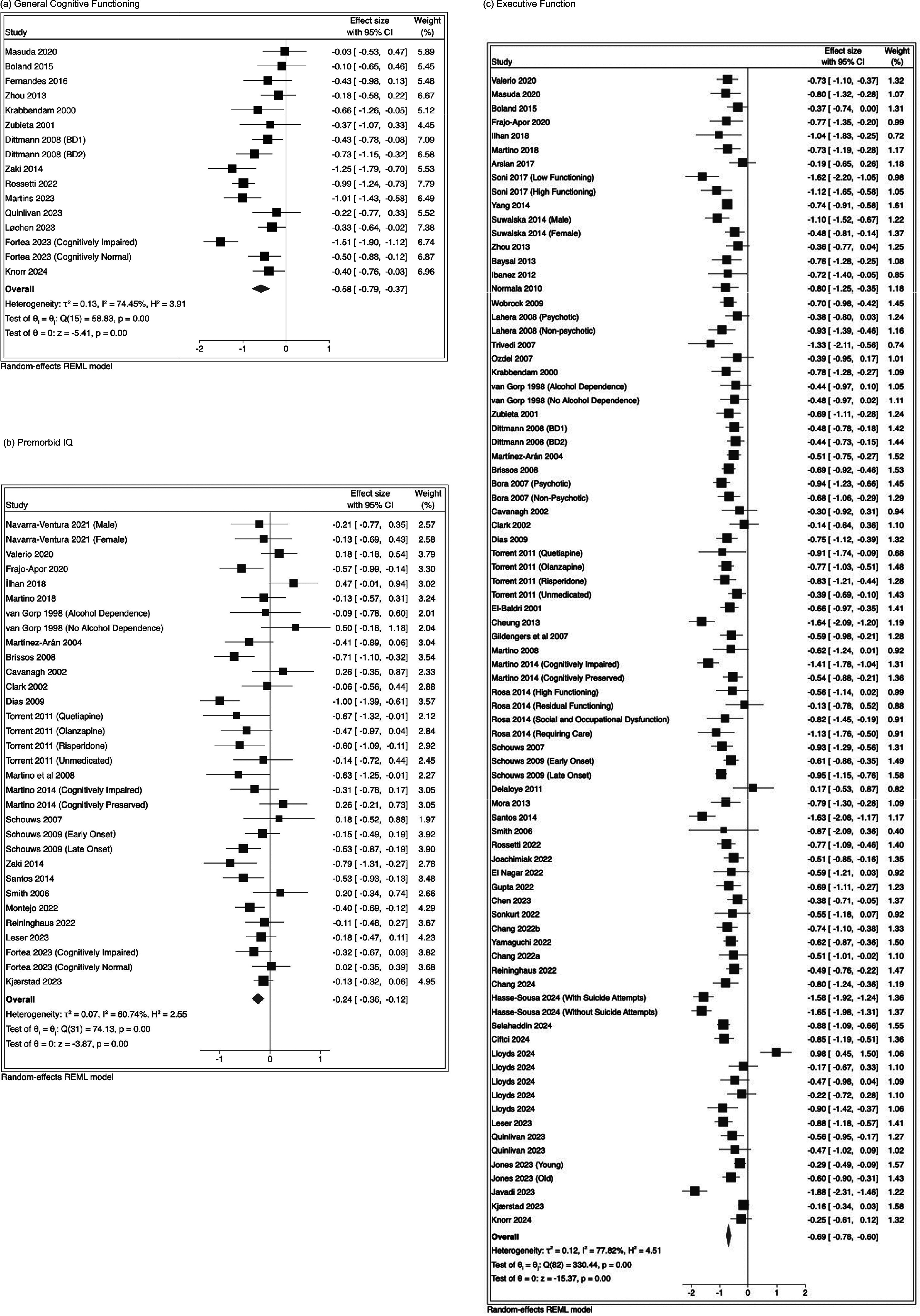
Forest plots showing the main effect of group (BD vs. HC) for general cognitive functioning, premorbid IQ, and executive function.

**Figure 3.**
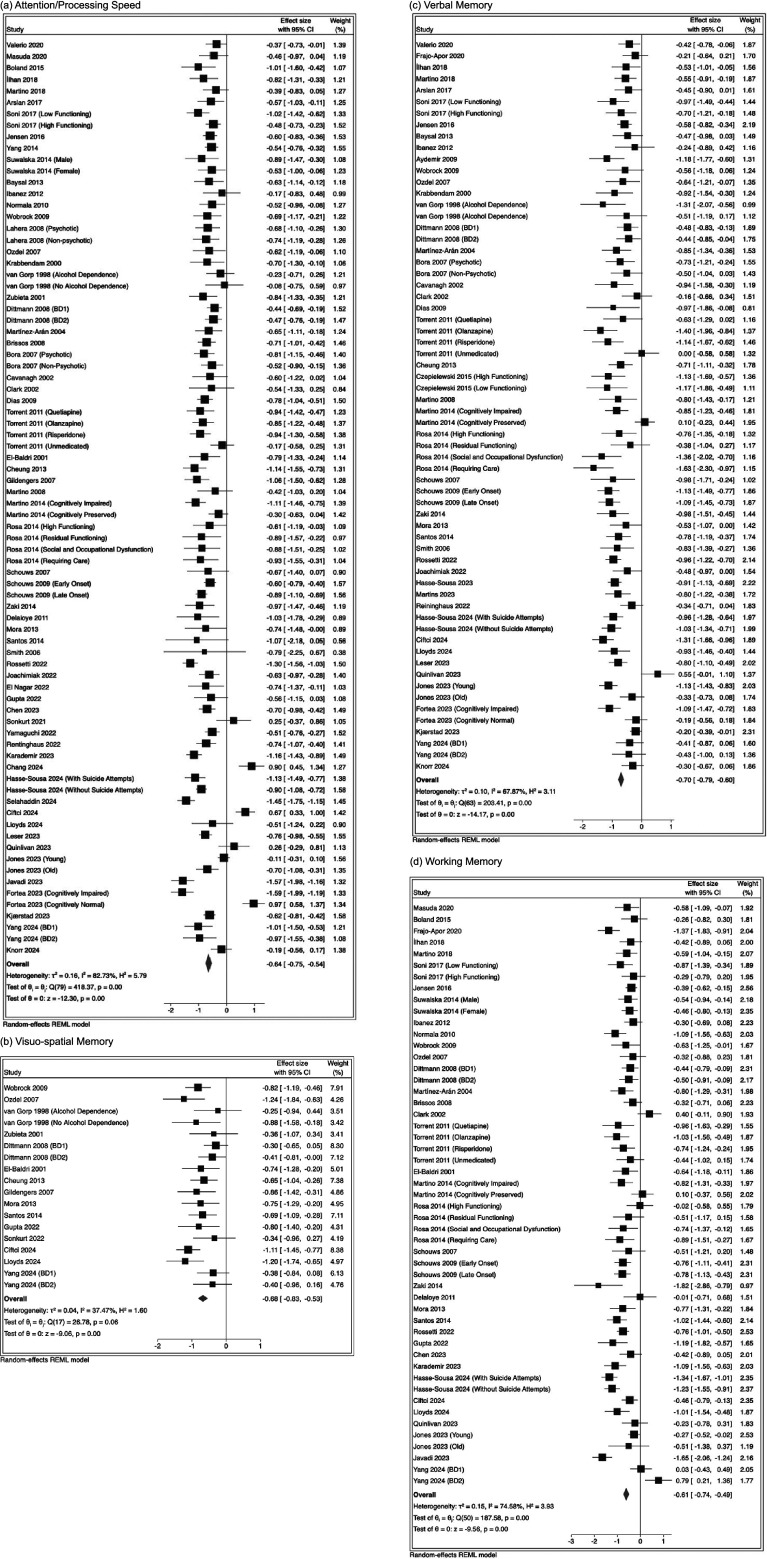
Forest plots showing the main effect of group (BD vs. HC) for verbal memory, visuo-spatial memory, working memory, and attention/processing speed.

Meta-analyses for each domain are presented in Table 3, with forest plots in Figures 2 and 3. Negative Hedge’s *g* effect sizes indicate worse performance in BD versus HC. All domains were found to be statistically significant. Funnel plots and Egger’s and Begg’s test statistics are presented in Supplementary Material F, showing no indication of publication bias.

**Table 3.** Results from meta-analyses and meta-regressions

		General cognitive functioning	Premorbid IQ	Executive Functioning	Working Memory	Verbal Memory	Visuo-spatial Memory	Attention/processing speed
Meta-analysis								
	Effect size (95%CI)	−.58** (−.79, −.37)	−.24 (−.36, −.12)	−.69** (−.76, −.60)	−.61** (−.74, −.49)	−.70** (−.80, −.60)	−.68** (−.83, −.53)	−.64** (−.75, −.54)
	N (studies)	16	32	83	51	64	18	80
	*I* ^2^ (%)	74.45	60.74	77.82	74.58	67.87	37.47	82.73
Meta−regressions								
	Premorbid IQ	.86		0.31	.78	.51	−0.15	.51
	Female Gender	−0.012	0.0015	−0.005	0.0059	0.0049	−0.0017	0.0029
	Age	0.003	−0.0014	0.011	−0.0065	−0.0062	0.032	−0.00002
	Education Years	0.028	0.0277	−0.013	0.039	.066*		0.01
	Age of Onset	−0.033	−0.011	−.0096	−0.013	−0.012	0.019	−0.0077
	Duration of Remission Duration			−0.0002	−0.0059	−.014	−0.015	0.041
	of Illness	0.0016	0.00053	−0.0026	−0.0005	0.00031	0.00058	0.00074
	Bipolar 1%	0.0056	−0.0024		−0.00061	−0.0056	0.0017	−0.0032
	Number of Episodes	0.034	−0.012	0.00055	−0.0017	0.0019	.032	−0.0009
	Number of depressive episodes	0.027	0.0041	0.014	−0.011	−0.041	0.012	0.0054
	Number of hypo manic episodes	0.02	−0.013	−0.0018	−0.011	0.038	0.0063	−0.0053
	Number of hospitalisations		0.018	−0.042	−0.057	−0.11	0.013	−0.062
	History of Psychosis	0.0057	0.00031	−0.00058	−0.0003	−0.0051	0.0025	−0.0013
	YMRS	−0.015	−0.15	0.034	−.25	−0.17	−0.043	−0.097
	HAM-D	0.015	0.035	0.04	0.093	0.059	−0.16	.063
	Mood Stabilisers	−0.021	0.0015	−0.0047	0.0014	0.002		
	Lithium	0.015	−0.0022	0.0014	0.0054	−0.002	0.0046	0.0058
	Anticonvulsants Antipsychotics	−0.015	−0.0074	−0.0044	−0.0039	0.0003	−0.011	0.0067
	Antidepressants	−0.0041	−0.0062	−0.002	−0.0057	−.0064	0.0012	−0.0048
	Benzodiazepines	0.015	0.006	0.0019	−0.0001	0.0018	−0.001	0.0025
	No medication	0.05	0.014	.017	0.0062	0.0057		0.041
		−0.0082	0.0034	0.0045	0.0021	.0099		0.0076

*Note*: Reporting the predictors of domain-specific cognitive functioning (measures of effect are *r* scores *p <.05, **p <.01. *p* values are adjusted following Benjamin–Hochberg (BH) correction.

The effect size between BD and HC in general cognitive functioning was significant after BH correction for general cognitive functioning (Hedge’s *g* = −0.58, 95% CI: −0.79, −0.37, *p* <.01 [*k* = 16, *I* ^2^ = 74.45%]). The largest domain-specific effect size was on verbal memory (Hedges *g* = −0.70, 95% CI: −0.79, −0.60, *p* <.01 [number of study groups (*k*) = 64, *I* ^2^ = 67.87%]); followed by executive function (Hedge’s *g* = −0.69, 95% CI: −0.78, −0.60, *p* <.01 [*k* = 83, *I* ^2^ = 77.82%]); visuo-spatial memory (Hedges *g* = – 0.68, 95%CI: −0.83, −0.53, *p* <.01 [*k* = 18, *I* ^2^ = 37.47%]); attention/processing speed (Hedge’s *g* = –0.64, 95%CI: −0.75, −0.54, *p* <.01 [*k* = 80, *I* ^2^ = 82.73%]) and working memory (Hedge’s *g* = −0.61, 95% CI: −0.74, −0.49, *p* <.01 [*k* = 67, *I* ^2^ = 74.58%]). A smaller effect size between groups was found for pre-morbid IQ (Hedge’s *g* = −0.24, 95% CI: −0.36, −0.12, *p* <.01 [*k* = 32, *I* ^2^ = 60.74%]).

### Associations of cognitive performance

Data from all 95 groups were included in the meta-regression analyses. Results are presented in Table 3. Prior to BH correction, there was a significant association between higher premorbid IQ and less impairment in working memory, β = .78, *p* <.01, less impairment in verbal memory, β = .51, *p* < .05, and less impairment in attention/processing speed, β = .51, *p* < .05. These associations did not remain significant following BH correction, with only more years of education being the correlate of lower impairment in verbal memory, β = .066, adjusted *p* < .05.

Higher premorbid IQ was associated with fewer manic episodes, β = −.054, *p* <.05. Better executive functioning was associated with longer duration of current remission, β = −.023, *p* < .05. Higher working memory was associated with lower antipsychotic use, β = −.0071, *p* <.05. Better verbal memory was associated with bipolar 2 diagnosis, β = −.0052, *p* <.05, and lower number of hospitalisations, β = −.12, p < .05. Lower visuospatial memory was associated with higher antidepressant use, β = −.43, *p* <.05. Better processing speed was associated with lower number of hospitalisations, β < −.0077, *p* <.05.

### Sensitivity analysis

Sensitivity analyses were conducted, removing studies that utilised ‘undefined’ cognitive assessments The largest effect size between BD and HC in cognitive performance was on executive function (Hedges *g* = .71, CI: −.79, −.63 [number of study groups = 79, *I* ^2^ = 69.78%]); followed by working memory (Hedges *g* = −.61, CI: −.74, −.49 [number of study groups = 49, *I* ^2^ = 74.72%]); and attention/processing speed (Hedges *g* = .64, CI: .74, .54 [number of study groups = 78, *I* ^2^ = 81.89%]).

## Discussion

The current meta-analysis examined generalised and domain-specific cognitive functioning in euthymic BD, updating previous reviews, examining a wider range of associative factors (Bourne et al., 2013; Man-Wrobel, Carreno & Dickinson, 2011). Cognitive performance was impaired to a similar degree across all domains studied, including executive functioning, verbal memory, attention, visuo-spatial memory, and general cognitive functioning, consistent with an earlier meta-analysis (Bourne et al., 2013). Impairment in premorbid IQ was lower yet statistically significant, partially supporting both neurodevelopmental and neurodegenerative theories. Cognitive performance remained largely unaccounted for by clinical and demographic variables, despite possible cumulative effects of these factors (Tsapekos, Strawbridge, Cella, Wykes, & Young, 2021).

### Cognitive decline in euthymic bipolar disorder

The significant impairment in premorbid IQ, partially supports a neurodevelopmental trajectory for at least a proportion of people. However, the extent of premorbid impairment was substantially smaller than in other domains at the group level, indicating possible neuroprogressive decline for another subgroup. This is consistent with a model suggesting cognitively distinct trajectories within the BD population (Millett & Burdick, 2021). Some longitudinal evidence indicates a decline in a subgroup in BD (up to 48%) (Hinrichs et al., 2017). Other reviews indicate some studies find no longitudinal decline (Bora & Özerdem, 2017; Martino et al., 2015). Nevertheless, longitudinal follow-up is often short (averaging 1-5 years), which may not be sufficient to detect decline (Millett & Burdick, 2021). Populations in our systematic review had a mean illness duration longer than 17 years, indicating established illness (Kim et al., 2015). Although general cognitive functioning was the second least impaired domain, small differences and large heterogeneity warrant caution in assuming that these results support evidence of greater impairment in specific domains (Bourne et al., 2013).

### Associations with cognitive impairment in euthymic bipolar disorder

Premorbid IQ explained considerable variance (i.e., large coefficient) in several cognitive domains, including working memory (β = .78), verbal memory (β = .51), and attention/processing speed (β = .51), although these did not remain significant following BH correction. Premorbid IQ did not significantly predict variance in other domains, or general cognitive functioning, likely explained by a lack of studies that reported data on both premorbid IQ and those domains. This was particularly the case in general cognitive functioning, where only three studies reported both, leading to insignificance, although the coefficient was large (β = .86) (Tsapekos et al., 2020).

Furthermore, considerable variation was left unexplained in cognitive domains, indicating the importance of determining associative factors other than premorbid IQ. Meta-regressions did not indicate any demographic or clinical predictors of cognitive performance, potentially warranting focus on other variables, or determining alternative methods to better detect the association of these variables. After correction, only higher education years significantly predicted higher verbal memory in BD compared to HC, which is perhaps unsurprising as verbal memory is acquired early in cognition, which is reflected in years of early education (Schneider, Knopf, & Sodian, 2010). Nevertheless, the coefficient of the association was small (β = .066).

In light of the foregoing results, the absence of significant demographic or clinical moderators beyond years of education (despite our a priori expectation that multiple factors would accentuate cognitive deficits), only education was identified as a significant moderator of cognitive performance. This possibly reflects methodological limitations inherent to study-level meta-regression. When cohort means, such as the average manic-episode count, are regressed on pooled effect sizes, genuine within-person associations are vulnerable to ecological bias and may be attenuated or reversed once data are aggregated across heterogeneous samples (Pollet, Stulp, Henzi, & Barrett, 2015). This bias is further compounded by variability in how primary studies operationalised each predictor, collinearity among illness-history indicators, and the loss of statistical power that accompanies covariates reported by only a subset of included investigations. Collectively, these factors are liable to skew relationships documented at the study level, possibly leaving only the modest association between educational attainment and verbal memory observable at the meta-analytic level.

Clarifying whether psychosis history, lithium exposure, episode burden and the remaining hypothesised variables genuinely moderate cognitive outcomes will therefore require participant-level methodologies. Individual-data or federated mega-analyses, together with harmonised prospective cohorts, will permit multilevel modelling that partitions variance within individuals, within studies, and between studies; thereby maximising statistical power while minimising ecological bias (Wakefield, 2009). These approaches are best suited to delineate the clinical and demographic determinants of cognitive trajectories in euthymic bipolar disorder.

### Limitations

Heterogeneity was observed in several domains, particularly attention/processing speed and executive functioning, warranting caution in the interpretation of comparably small differences in effect size between domains. Although the current review benefited from having samples from several countries, differing levels of functioning (ranging from high functioning to being unable to maintain personal self-care), substance use, BD type and suicidality, add to this heterogeneity, which may have obscured an effect.

Heterogeneity may be explained by evidence of cognitive clusters in BD (i.e., severe impairment across domains, selective impairment in specific domains, and intact cognitive functioning) (Burdick et al., 2014; Tsapekos et al., 2020), which could not be addressed in the group level comparisons we conducted. Nevertheless, our results are broadly in keeping with those of Bourne et al. (2013), with greater effect sizes observed in this study.

Group-level comparisons may explain why some specific clinical and demographic factors were not associated with cognitive impairment, as seen in individual studies.

Another limitation is the categorising of cognitive tests into domains. Although there is strong evidence of the utility of this (Baune & Malhi, 2015), some assessments use skills from multiple domains, leading to difficulty in the choice of which domain to use for each test. Other ways of assessing cognitive functioning include studies classifying samples in homogeneous cognitive subgroups using data-driven approaches (Burdick et al., 2014; Tsapekos et al., 2020), suggesting different levels of impairment (i.e., no impairment, impairment in certain domains, and impairment across domains).

On a related note, the use of cross-sectional data means causal inference is difficult, with very few longitudinal studies existing in the literature, indicating a decline following the first episode (Zanelli, 2012; Zanelli et al., 2019), warranting future focus on longitudinal studies. Nevertheless, the Bipolar Commission found that BD is on average diagnosed 9.5 years after illness onset (Goodwin et al., 2022), indicating the need for researchers to determine alternative ways of following up with individuals at-risk of later BD diagnosis, as once diagnosed, decline may have already occurred (Zanelli, 2012).

Finally, a recent analysis of a large cohort (McCutcheon, Keefe, McGuire, & Marquand, 2024), found that cognitive impairment across psychotic disorders (including BD) may be related to risk factor exposure (i.e., different exposure to HC) as opposed to disease-specific effects. This suggests more scrutiny of control groups for risk factor exposure, which was not possible in the current analysis, as a lot of these were not reported.

### Implications and future directions

The high prevalence and severity of impairment across domains of cognitive functioning warrant increased focus for both understanding the nature and nuances of this impairment and increasing the provision of interventions to tackle cognitive difficulties. For the former, longitudinal studies will help delineate subgroups with differential cognitive trajectories. Optimally, these studies will focus on those at high risk of developing BD or first-episode psychosis (Keramatian, Torres, & Yatham, 2021) and people with first-episode mania (Jauhar et al., 2019). This may facilitate the delivery of targeted interventions at an early stage, with the aim of not only restoring potential deficits but also preventing/slowing further decline in cognitive performance (Miskowiak et al., 2022). CR aims to improve cognitive functioning through enhancing metacognitive skills, developing compensatory strategies, and training executive functioning. Initial evidence suggests it may be effective in tackling cognitive difficulties and transferring cognitive gains into functional improvement in euthymic BD (Strawbridge et al., 2021; Tsapekos et al., 2023; Tsapekos, Strawbridge, Cella, Wykes, & Young, 2022). Larger trials are currently underway to assess the efficacy and potential mechanisms of this treatment paradigm (Tsapekos et al., 2023). However, the intervention has not yet been tested specifically at early stages of the illness, which surely represents a promising future research direction.

## Conclusion

The present systematic review and meta-analysis indicates moderate (>0.5 SD below the mean of HC) cognitive impairment in specific domains (executive function, working memory, verbal memory, visuo-spatial memory and attention/processing speed) and mild impairment (<0.5 SD below the mean of HC) in general cognitive function, in BD, consistent with previous findings of deficits across domains (Bourne et al., 2013). Comparably lower impairment in premorbid IQ provides some basis for both neurodevelopmental and neuroprogressive hypotheses.

Nevertheless, heterogeneity was high across domains, which may be explained by cognitive clusters in BD (Burdick et al., 2014) and by potentially untested correlates (e.g., schizophrenia polygenic risk score (Ohi et al., 2023; Wu et al., 2024) and family history, (Landau, Raymont, & Frangou, 2003). Future research should determine reasons for heterogeneity in longitudinal analyses to tailor future treatments, such as CR, for individuals at risk of poor cognitive and functional outcomes.

## Supporting information

Swidzinski et al. supplementary material Swidzinski et al. supplementary material

## Supplementary material

The supplementary material for this article can be found http://doi.org/10.1017/S0033291725101827.
