# Supplementary material for: Domain-specific cognitive function in euthymic bipolar disorder: a systematic review and meta-analysis
Source: Psychol Med. 2025 Nov 5;55:e336. doi: 10.1017/S0033291725101827 (PMC13058641; doi:10.1017/S0033291725101827)
Supplement: Swidzinski et al. supplementary material [file S0033291725101827sup001.pdf]

Supplementary Material A: Prisma Checklist

Section and Topic	Item #	Checklist item	Location where item is reported
**TITLE**			
Title	1	Identify the report as a systematic review.	Page 1
**ABSTRACT**			
Abstract	2	See the PRISMA 2020 for Abstracts checklist.	Page 2
**INTRODUCTION**			
Rationale	3	Describe the rationale for the review in the context of existing knowledge.	Page 3-4
Objectives	4	Provide an explicit statement of the objective(s) or question(s) the review addresses.	Page 4
**METHODS**			
Eligibility criteria	5	Specify the inclusion and exclusion criteria for the review and how studies were grouped for the syntheses.	Page 5
Information sources	6	Specify all databases, registers, websites, organisations, reference lists and other sources searched or consulted to identify studies. Specify the date when each source was last searched or consulted.	Page 5
Search strategy	7	Present the full search strategies for all databases, registers and websites, including any filters and limits used.	Page 5
Selection process	8	Specify the methods used to decide whether a study met the inclusion criteria of the review, including how many reviewers screened each record and each report retrieved, whether they worked independently, and if applicable, details of automation tools used in the process.	Page 5-7
Data collection process	9	Specify the methods used to collect data from reports, including how many reviewers collected data from each report, whether they worked independently, any processes for obtaining or confirming data from study investigators, and if applicable, details of automation tools used in the process.	Page 5-6
Data items	10a	List and define all outcomes for which data were sought. Specify whether all results that were compatible with each outcome domain in each study were sought (e.g. for all measures, time points, analyses), and if not, the methods used to decide which results to collect.	Page 6-7
	10b	List and define all other variables for which data were sought (e.g. participant and intervention characteristics, funding sources). Describe any	Page 5-7

Section and Topic	Item #	Checklist item	Location where item is reported
		assumptions made about any missing or unclear information.	
Study risk of bias assessment	11	Specify the methods used to assess risk of bias in the included studies, including details of the tool(s) used, how many reviewers assessed each study and whether they worked independently, and if applicable, details of automation tools used in the process.	Page 5-6
Effect measures	12	Specify for each outcome the effect measure(s) (e.g. risk ratio, mean difference) used in the synthesis or presentation of results.	Page 6
Synthesis methods	13a	Describe the processes used to decide which studies were eligible for each synthesis (e.g. tabulating the study intervention characteristics and comparing against the planned groups for each synthesis (item #5)).	Page 5-6
	13b	Describe any methods required to prepare the data for presentation or synthesis, such as handling of missing summary statistics, or data conversions.	Page 5-7
	13c	Describe any methods used to tabulate or visually display results of individual studies and syntheses.	Page 5-7
	13d	Describe any methods used to synthesize results and provide a rationale for the choice(s). If meta-analysis was performed, describe the model(s), method(s) to identify the presence and extent of statistical heterogeneity, and software package(s) used.	Page 5-7
	13e	Describe any methods used to explore possible causes of heterogeneity among study results (e.g. subgroup analysis, meta-regression).	Page 7
	13f	Describe any sensitivity analyses conducted to assess robustness of the synthesized results.	Page 7
Reporting bias assessment	14	Describe any methods used to assess risk of bias due to missing results in a synthesis (arising from reporting biases).	Page 5-7
Certainty assessment	15	Describe any methods used to assess certainty (or confidence) in the body of evidence for an outcome.	Page 5-7
**RESULTS**			
Study selection	16a	Describe the results of the search and selection process, from the number of records identified in the search to the number of studies included in the review, ideally using a flow diagram.	Figure 1
	16b	Cite studies that might appear to meet the inclusion criteria, but which were excluded, and explain why they were excluded.	Page 8

Section and Topic	Item #	Checklist item	Location where item is reported
Study characteristics	17	Cite each included study and present its characteristics.	Page 8, Table 1, Table 2, Supplementary Material C & D
Risk of bias in studies	18	Present assessments of risk of bias for each included study.	Page 9
Results of individual studies	19	For all outcomes, present, for each study: (a) summary statistics for each group (where appropriate) and (b) an effect estimate and its precision (e.g. confidence/credible interval), ideally using structured tables or plots.	Page 9
Results of syntheses	20a	For each synthesis, briefly summarise the characteristics and risk of bias among contributing studies.	Supplementary Material C & D, Page 9
	20b	Present results of all statistical syntheses conducted. If meta-analysis was done, present for each the summary estimate and its precision (e.g. confidence/credible interval) and measures of statistical heterogeneity. If comparing groups, describe the direction of the effect.	Page 9
	20c	Present results of all investigations of possible causes of heterogeneity among study results.	Page 9, Table 3
	20d	Present results of all sensitivity analyses conducted to assess the robustness of the synthesized results.	Page 9
Reporting biases	21	Present assessments of risk of bias due to missing results (arising from reporting biases) for each synthesis assessed.	Page 9, Supplementary Material F
Certainty of evidence	22	Present assessments of certainty (or confidence) in the body of evidence for each outcome assessed.	Page 9
**DISCUSSION**			

Section and Topic	Item #	Checklist item	Location where item is reported
Discussion	23a	Provide a general interpretation of the results in the context of other evidence.	Page 10
	23b	Discuss any limitations of the evidence included in the review.	Page 11
	23c	Discuss any limitations of the review processes used.	Page 11
	23d	Discuss implications of the results for practice, policy, and future research.	Page 11
**OTHER INFORMATION**			
Registration and protocol	24a	Provide registration information for the review, including register name and registration number, or state that the review was not registered.	Page 5
	24b	Indicate where the review protocol can be accessed, or state that a protocol was not prepared.	Page 5
	24c	Describe and explain any amendments to information provided at registration or in the protocol.	Page 5
Support	25	Describe sources of financial or non-financial support for the review, and the role of the funders or sponsors in the review.	No support external to coauthors.
Competing interests	26	Declare any competing interests of review authors.	N/A
Availability of data, code and other materials	27	Report which of the following are publicly available and where they can be found: template data collection forms; data extracted from included studies; data used for all analyses; analytic code; any other materials used in the review.	Availability of tables, figures and supplementary materials made clear throughout the manuscript

From: Page MJ, McKenzie JE, Bossuyt PM, Boutron I, Hoffmann TC, Mulrow CD, et al. The PRISMA 2020 statement: an updated guideline for reporting systematic reviews. *BMJ 2021*;. doi: 10.1136/bmj.n71

Supplementary Material B: Newcastle Ottawa Scale for Quality Assessment Scoring Process Explanation

Selection: (Maximum 5 scores)

- **Representativeness of the sample (/1):**
    - Truly representative of the average in the target population (all subjects or random sampling)- 1 score
    - Somewhat representative of the average in the target group (non-random sampling)- 1 score
    - Selected group of users/convenience sample. - 0 score
    - No description of the derivation of the included subjects. - 0 score
- **Sample size (/1):**
    - Have at least 50 participants for each of the group based on design or analysis. – 1score
    - Have less than 50 participants for one or few groups in the study. - 0 score
- **Non-respondents Bias (/1):**
    - Provided information or basic summary of non-respondent characteristics. – 1 score
    - No information provided. – 0 score

- **Ascertainment of Euthymic Biploar (/2):**
  - Used Scheduled Clinical Interview of DSM-V or similar structed scheduled clinical interview. – 1 score
  - Used Validated measurement tool for depression and mania, such as HAM-D, Yang Mania Rating Scale or equivalent of such scales. – 1 score
  - Validated measurement methods for symptoms of bipolar disorder not described. – 0 score

Comparability: (Maximum 2 scores)

- **Comparability of subjects in different groups based on design or analysis.**
  - Age was comparable between the groups. – 1 score
  - Years of Education or Education level were comparable between the groups. – 1 score
  - Information was not provided, or groups were not comparable. – 0 score

Outcome: (Maximum 3 scores)

- **Assessment of the outcome (/2)**
  - Outcomes are assessed using validated tests and being stated clear. – 2 scores
  - Outcomes are assessed using validated tests and stated which tests but also mentioning unstated test or not explaining what test used. – 1 score
  - Outcomes are reported without stating what test used or no information provided. – 0 score

- **Statistical test (/1):**
    - Statistical test used to analyze the data clearly described, appropriate, and measures of association presented including confidence intervals and probability level (P value). – 1 score
    - Statistical test not appropriate, not described, or incomplete. – 0 score

Cross-sectional Studies: Very Good Studies: 9-10 points Good Studies: 7-8 points Satisfactory Studies: 5- 6 points Unsatisfactory Studies: 0 to 4 points

Supplementary Material C: Newcastle Ottawa Scale Quality Assessment Scores for Cross-sectional studies

Cross-sectional studies	Selection (S) Representativeness of the sample to BD and HC groups (/1)	Sample size (> 50 participants per group) (/1)	Non-response bias (/1)	Ascertainment of euthymic BD (/2)	Comparability (C) Comparability of groups (/2)	Outcome (O) Assessment of outcome statistical test (/3)	Conclusion
1) Navarra-Ventura et al 2021	1	0	0	1	2	3	Good
2) Valerio et al 2020	1	1	0	2	2	2	Good
3) Masuda et al 2020	0	0	0	2	1	2	Fair
4) Boland et al 2015	1	0	0	2	2	2	Good
5) Frajo-Apor et al 2020	1	0	0	2	1	2	Fair
6) İlhan et al 2018	1	0	0	2	2	3	Good
7) Martino et al 2018	0	0	0	2	2	3	Good
8) Arslan et al 2017	1	0	0	2	2	3	Good
9) Soni et al 2017	1	0	0	2	2	3	Good
10) Jensen et al 2016	1	1	0	2	2	2	Good
11) Fernandes et al 2016	1	0	0	2	2	3	Good
12) Yang et al 2014	1	1	0	2	2	3	Very Good
13) Suwalska et al 2014	1	1	0	2	2	3	Very Good
14) Zhou et al 2013	1	0	0	2	1	3	Good
15) Baysal et al 2013	1	0	1	2	2	3	Very Good
16) Ibanez et al 2012	0	0	0	2	2	2	Fair
17) Normala et al 2010	1	0	0	2	1	3	Good
18) Aydemir et al 2009	0	0	1	2	2	2	Good
19) Wobrock et al 2009	1	0	0	1	1	3	Fair
20) Lahera et al 2008	1	0	0	2	2	3	Good
21) Trivedi et al 2007	1	0	0	2	2	3	Good
22) Ozdel et al 2007	0	0	0	2	2	3	Good
23) Krabbendam et al 2000	1	0	1	2	1	3	Good
24) van Gorp et al 1998	1	0	0	2	2	3	Good
25) Zubieta et al 2001	1	0	0	2	2	3	Good
26) Dittmann et al 2008	0	0	0	2	1	2	Fair
27) Martínez-Arán et al 2004	0	0	0	2	2	3	Good

28)	Brissos et al 2008	0	1	1	1	2	3	Good
29)	Bora et al 2007	0	0	0	2	2	3	Good
30)	Cavanagh et al 2002	1	0	1	2	1	3	Good
31)	Clark et al 2002	0	0	0	2	2	2	Fair
32)	Dias et al 2009	0	1	0	2	0	3	Fair
33)	Torrent et al 2011	1	0	0	1	0	3	Fair
34)	El-Badri et al 2001	1	0	0	1	1	3	Fair
35)	Cheung et al 2013	0	1	1	2	1	2	Good
36)	Czepielewski et al 2015	1	0	0	2	2	3	Good
37)	Gildengers et al 2007	0	0	0	1	1	2	Poor
38)	Martino et al 2008	0	0	0	2	2	3	Good
39)	Martino et al 2014	1	0	0	2	2	3	Good
40)	Rosa et al 2014	0	0	0	1	0	2	Poor
41)	Schouws et al 2007	1	0	0	2	2	2	Good
42)	Schouws et al 2009	1	1	0	2	1	3	Good
43)	Zaki et al 2014	1	0	0	2	2	2	Good
44)	Smith 2006	0	0	0	2	2	3	Good
45)	Gupta et al 2022	1	0	0	2	2	3	Good
46)	Rossetti et al 2022	1	1	0	2	1	3	Good
47)	Joachimiak et al 2022	1	0	0	1	2	3	Good
48)	El Nagar et al 2022	0	0	0	2	2	3	Good
49)	Chen et al 2023	1	0	0	1	2	3	Good
50)	Hasse-Sousa et al 2023	1	1	0	1	0	3	Fair
51)	Sonkurt et al 2022	0	0	0	2	2	3	Good
52)	Martins et al 2023	1	0	0	2	0	2	Fair
53)	Chang et al 2022a	1	1	0	2	1	3	Good
54)	Mentejo et al 2022	0	1	0	2	1	3	Good
55)	Yamaguchi et al 2022	1	1	1	1	1	3	Good
56)	Chang et al 2022b	1	1	0	2	1	3	Good
57)	Reininghaus et al 2022	1	1	0	1	1	3	Good
58)	Karademir et al 2024	1	0	1	2	1	2	Good

59)	Chang et al 2024	1	0	0	2	1	2	Fair
60)	Hasse-Sousa et al 2024	1	1	0	1	0	2	Fair
61)	Selahaddin et al 2024	1	1	0	1	2	2	Good
62)	Cifci et al 2024	1	0	0	1	1	2	Fair
63)	Lloyds et al 2024	1	0	0	1	1	2	Fair
64)	Leser et al 2023	0	1	1	2	0	2	Fair
65)	Quinlivan et al 2023	1	0	0	2	2	2	Good
66)	Jones et al 2023	1	0	0	2	1	2	Fair
67)	Javadi et al 2023	1	1	0	1	1	2	Fair
68)	Løchen et al 2023	1	1	0	1	1	2	Fair
69)	Fortea et al 2023	1	1	1	2	0	2	Good
70)	Yang et al 2024	1	0	0	2	1	1	Fair

Supplementary Material D: Newcastle Ottawa Quality Assessment Scores for Longitudinal Studies

	Selection (S)			Comparability (C)		Outcome (O)			Conclusion
Cohort study	Representativeness of the sample to genuine BD and HC groups (/1)	Sample size of cohort (/1)	Non-response bias (/1)	Ascertainment of euthymic BD (/2)	Comparability of groups (/2)	Assessment of outcome (cognitive tests used) (/2)	Follow-up long enough (/1)	Adequacy of follow-up of cohorts (/1)	
1) Delaloye et al 2011	1	0	0	1	2	2	1	0	Fair
2) Mora et al 2013	0	0	1	1	2	2	1	1	Good
3) Santos et al 2014	0	0	0	2	1	2	1	0	Fair
4) Kjærstad et al 2023	1	1	0	1	1	2	1	0	Fair
5) Knorr et al 2024	0	0	0	1	2	1	1	0	Poor

Supplementary Material E: Neurocognitive assessments broken down into cognitive domains

Domain	Battery	Abbreviation	Foreign or Other Equivalents	Scoring Techniques	Examples of studies that use each measure
General cognitive ability	Wechsler Adult Intelligence Scale Full-Scale IQ	WAIS-III/ IV/ R	HAWIE<br>GIT	IQ Score	Navarra-Ventura et al., 2021; Ditmmann et al., 2008; Krabbendam et al., 2020
	Wechsler Abbreviated Scale of Intelligence	WASI		IQ Score	Fernandes et al., 2016
	Shipley IQ			IQ Score	Ozdel et al., 2001
	Ravens Matrices			Percentile	Zhou et al., 2013
	Cambridge Neurological Test Automated Battery	CANTAB			Wobrock et al., 2009
	Kauffman Brief Intelligence Test II	KBIT-II		Percentile	Hays et al., 2002
	Unstated general cognitive ability				Masuda et al., 2020
Premorbid	National Adult Reading Test	NART		IQ Score	van Gorp et al., 1998
	Multiple Choice Vocabulary Intelligence Test	MWT-B			Frajo-Apor et al., 2020
	Information and Vocabulary Subsets of the Wechsler Adult Intelligence Scale	WAIS-R Vocab/Information			Dias et al., 2009
	Unstated premorbid IQ				Shouws et al., 2007
Executive function	Verbal Fluency Test	VFT		Phonological fluency test<br>Categorical fluency test	Santos et al., 2014; Martino et al., 2014
	Trail Making Task- B	TMT-B	Concept Shifting Test	Errors<br>Time taken (s)	İlhan et al., 2018
	Tower of London	ToL			Clark et al., 2002
	Tower of Hanoi	ToH			Clark et al., 2002

	Stockings of Cambridge	SOC		
	Intra/Extra-Dimensional Set-Shifting Performance	IED		
	Stroop (Incongruent Trial)		Inhibition/ Switching from DKEFS	
	Concept Shifting Test	CST		
	Consonant Updating Task	CUT		
	Brief Assessment of Cognition in Schizophrenia for Verbal Fluency	BACS Verbal Fluency		Frajo-Apor et al., 2020
	Wisconsen Card Sorting Task	WCST	Categories Completed<br>Number of perseverative errors<br>Number of non-perseverative errors	Martino et al., 2018
Working Memory	Digit Span Backwards (verbal and visual)		Number of digits recalled	Soni et al., 2016
	Letter-Number Sequencing Task			Rosa et al., 2014
	Working Memory Subset of the Wechsler Memory Scale	WMS-R		İlhan et al., 2018
	Working Memory Subset of the Test battery for attentiveness	TAP		Wobrock et al., 2009
	Spatial Working Memory Test	SWMT	Errors and Strategy	Trivedi et al., 2008
	Digit Sequencing			Frajo-Apor et al., 2020
	Unstated working memory			Jensen et al., 2016
Verbal Memory	Amsterdam Short Term Memory Test	ASTM		Schouws et al., 2017

	Verbal Learning Memory Tests	VLT	California Verbal Learning Test<br>Hopkins Verbal Learning Test<br>Regensburger-Wortflussigkeits-Test	Immediate recall<br>Delayed Recall<br>Recognition	Jensen et al., 2016; Aydemir et al., 2008; Torrent et al., 2011
	Signoret Memory Battery (SMB) for Verbal Memory	SMB			Valerio et al., 2020
	Verbal Memory Subsets of WMS-R, BACS, WAIS-R and RBANS				Lin et al ., 2017; Frajo-Apor et al., 2020; Dias et al., 2008
Visuo-Spatial Memory	Rey-Osterrieth Complex Figure Test (ROCFT)				Santos et al., 2014
	Visuo-spatial subset of the WMS-R and BACS				Ozdel et al., 2001
	CANTAB Spatial Recognition Memory				Wobrock et al., 2009
	Unstated Visuospatial Memory				Cheung et al., 2013
Attention/ Processing Speed	Trail Making Task A (TMT-A)			Trails A time taken in seconds	Dittmann et al., 2008
	Stroop Conrguent Trials				Ozdel et al., 2001
	Digit Forward (Verbal or Visual)				Ozdel et al., 2001
	Test Battery for Attentiveness (TAP) for Divided Attention				Wobrock et al., 2009
	CANTAB Rapid Visual Processing (RVP)				Masuda et al., 2020
	Test of Variables of Attention (TOVA)				Ozdel et al., 2001
	WAIS-III Digit Symbol				Lin et al., 2017
	Simple and Complex Motor Speed				Martino et al., 2008
	Manual Imitation Test (MIT)				Ozdel et al., 2001
	Symbol Digit Modalities Test (SDMT)				Santos et al., 2014
	Unstated Attention/ Processing Speed				Cheung et al., 2013

Supplementary Material F: Evaluation of Publication Bias through Funnel Plots and Egger`s and Begg`s Test Statistics

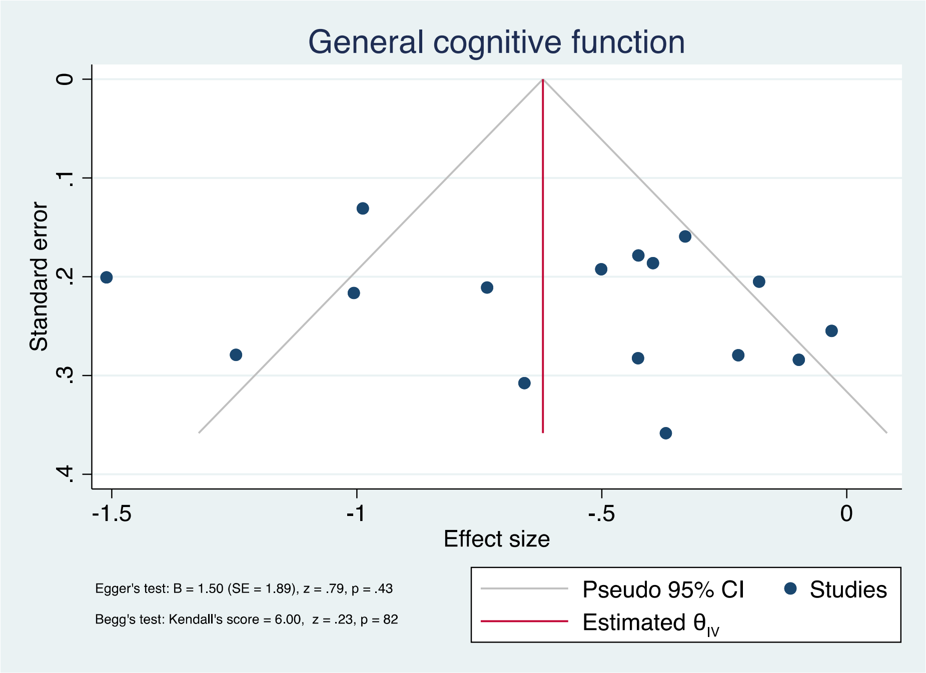

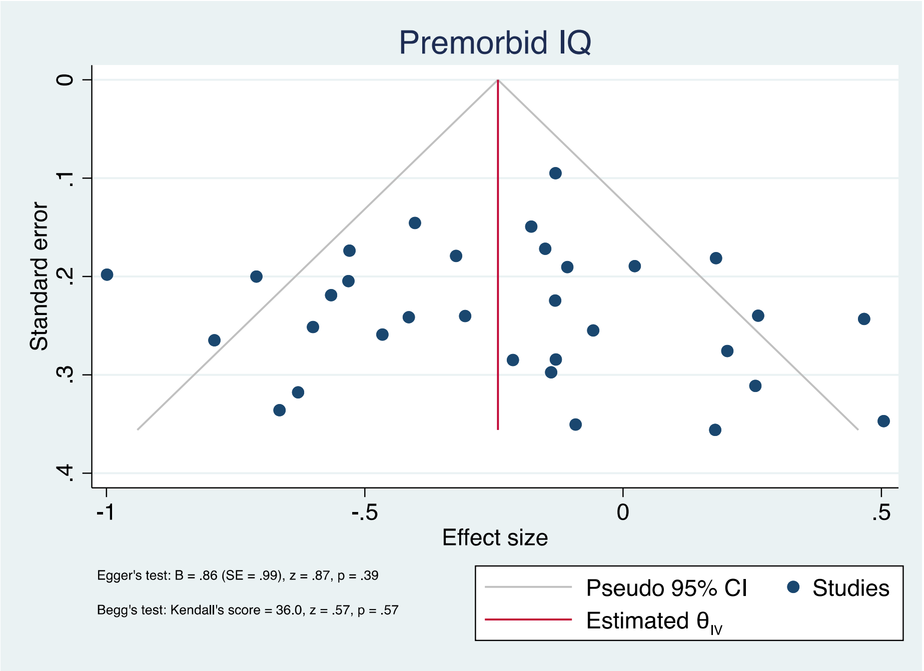

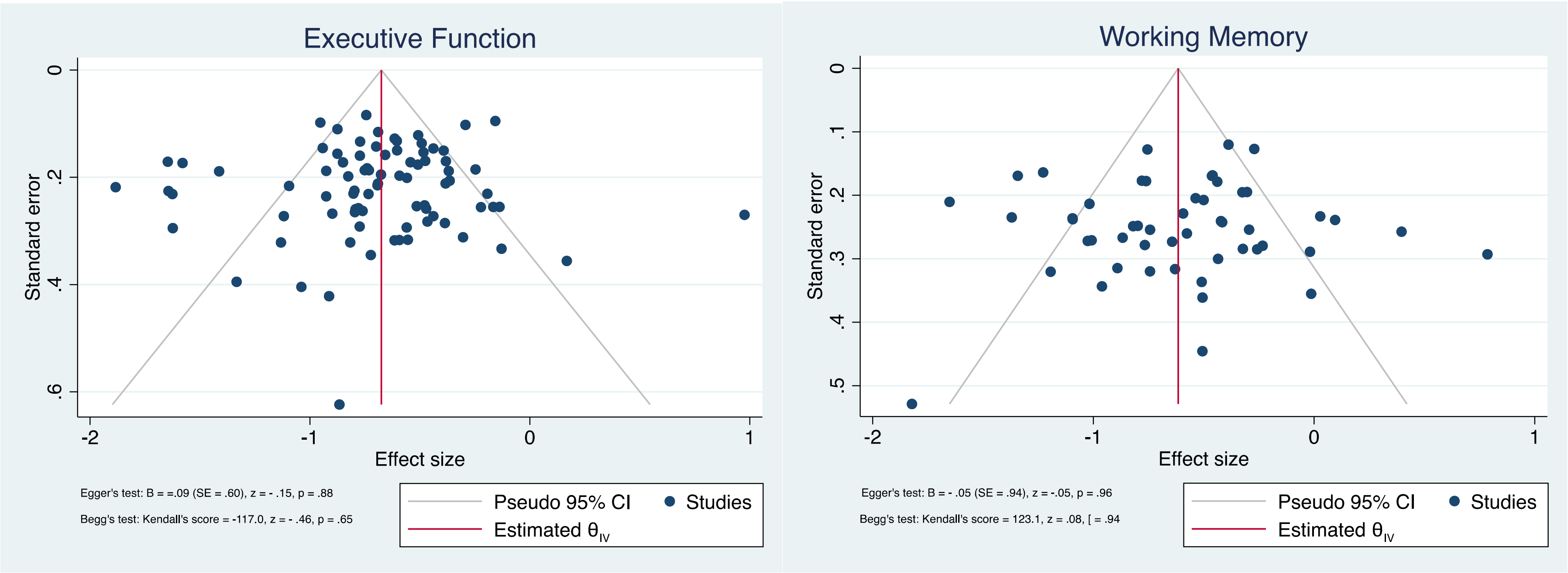
Executive Function
Standard error
0
.2
.4
.6
-2
-1
0
1
Effect size
Egger's test: B = =.09 (SE = .60), z = - .15, p = .88
Begg's test: Kendall's score = -117.0, z = - .46, p = .65
Pseudo 95% CI
Studies
Estimated θ_{IV}
Working Memory
Standard error
0
.1
.2
.3
.4
.5
-2
-1
0
1
Effect size
Egger's test: B = - .05 (SE = .94), z = -.05, p = .96
Begg's test: Kendall's score = 123.1, z = .08, [= .94
Pseudo 95% CI
Studies
Estimated θ_{IV}

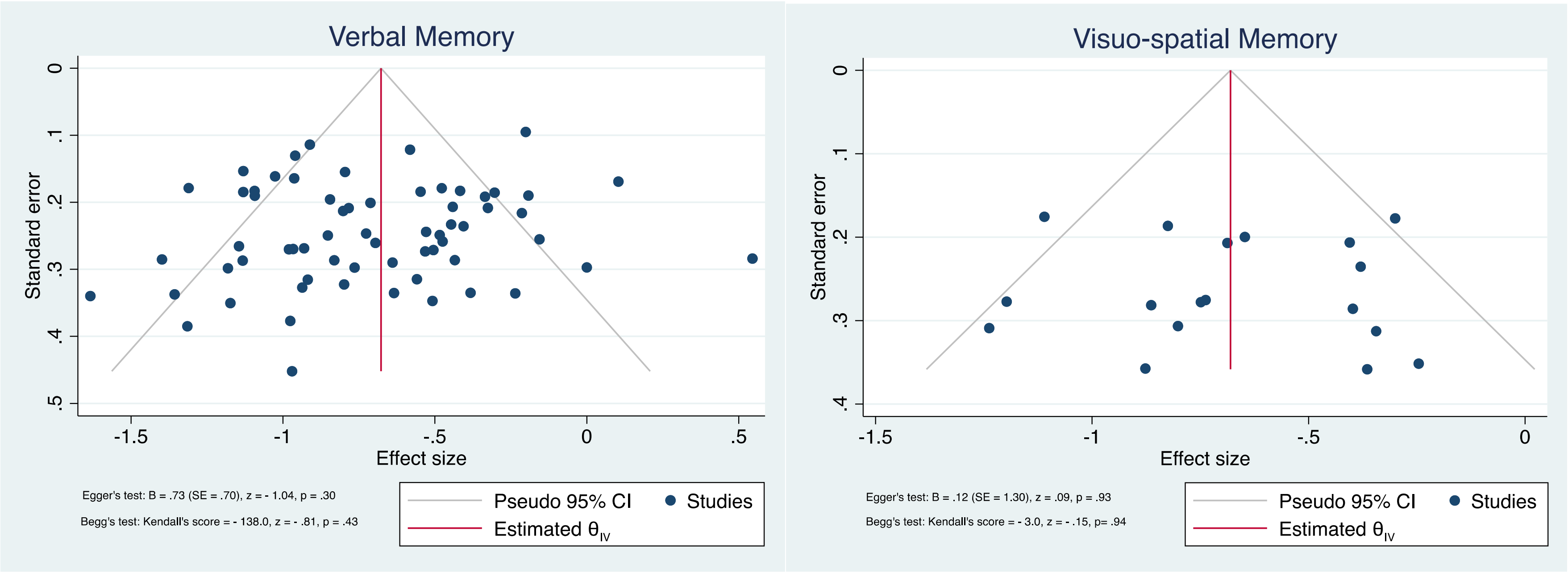
Verbal Memory
Standard error
0
.1
.2
.3
.4
.5
-1.5
-1
-.5
0
.5
Effect size
Egger's test: B = .73 (SE = .70), z = - 1.04, p = .30
Begg's test: Kendall's score = - 138.0, z = - .81, p = .43
Pseudo 95% CI
Studies
Estimated θ_{IV}
Visuo-spatial Memory
Standard error
0
.1
.2
.3
.4
-1.5
-1
-.5
0
Effect size
Egger's test: B = .12 (SE = 1.30), z = .09, p = .93
Begg's test: Kendall's score = - 3.0, z = - .15, p= .94
Pseudo 95% CI
Studies
Estimated θ_{IV}

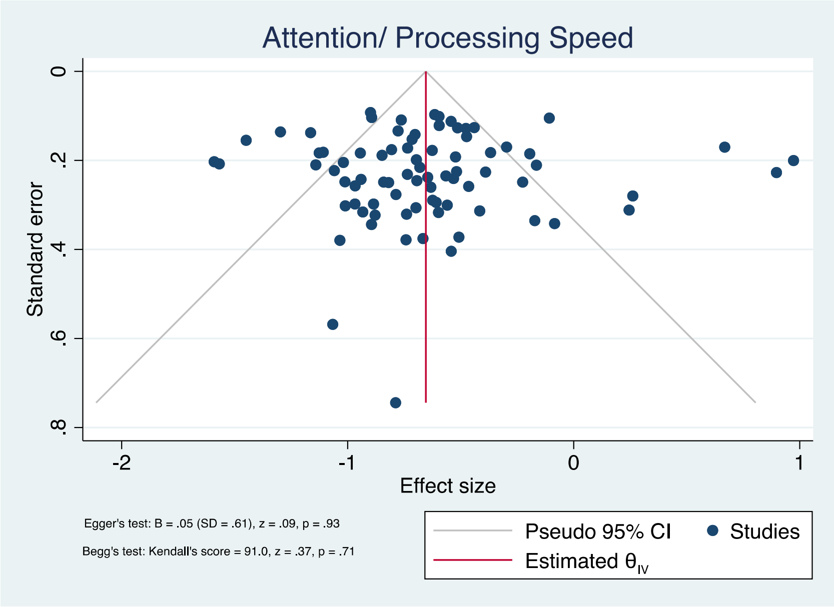
Attention/ Processing Speed
Standard error
0
.2
.4
.6
.8
-2
-1
0
1
Effect size
Egger's test: B = .05 (SD = .61), z = .09, p = .93
Begg's test: Kendall's score = 91.0, z = .37, p = .71
Pseudo 95% CI
Studies
Estimated θIV
